# Impact of Air Pollutants on Oxidative Stress in Common Autophagy-Mediated Aging Diseases

**DOI:** 10.3390/ijerph120202289

**Published:** 2015-02-17

**Authors:** Mohamed Saber Numan, Jacques P. Brown, Laëtitia Michou

**Affiliations:** 1 Department of Endocrinology and Nephrology, Centre Hospitalier Universitaire de Québec Research Centre, Québec, QC, G1V 4G2, Canada; E-Mail: mohamed.numan.1@ulaval.ca; 2 Division of Rheumatology, Department of Medicine, University Laval, Québec, QC, G1V 4G2, Canada; E-Mail: jacques.brown@crchul.ulaval.ca; 3 Department of Rheumatology, Centre Hospitalier Universitaire de Québec, Québec, QC, G1V 4G2, Canada

**Keywords:** Paget’s disease of bone, neurodegenerative diseases, autophagy, SQSTM1/p62, oxidative stress, cigarettes, tobacco smoke, wood heating, air pollutants, aging

## Abstract

Atmospheric pollution-induced cellular oxidative stress is probably one of the pathogenic mechanisms involved in most of the common autophagy-mediated aging diseases, including neurodegenerative diseases such as amyotrophic lateral sclerosis (ALS), Alzheimer’s, disease, as well as Paget’s disease of bone with or without frontotemporal dementia and inclusion body myopathy. Oxidative stress has serious damaging effects on the cellular contents: DNA, RNA, cellular proteins, and cellular organelles. Autophagy has a pivotal role in recycling these damaged non-functional organelles and misfolded or unfolded proteins. In this paper, we highlight, through a narrative review of the literature, that when autophagy processes are impaired during aging, in presence of cumulative air pollution-induced cellular oxidative stress and due to a direct effect on air pollutant, autophagy-mediated aging diseases may occur.

## 1. Introduction 

Forty-eight years ago, Deter and De Duve coined the term “autophagy” following their observation of degradation of mitochondria within rat liver lysosomes [1]. Autophagy means “self-eating” [2] and it attenuates the consequences of the damaging action of stress [3,4] resulting from oxidative stress [5] or starvation [6]. It provides energy to the cells to keep their metabolic pathways in a normal state [7]. Furthermore, autophagy helps the cell to get rid of non-functional or misfolded proteins and damaged non-functional organelles [8,9]. Autophagy pathway consists of four principal steps that begin by stimulation and initiation, followed by nucleation, elongation, and eventually maturation and formation of autolysosomes that result from fusion between autophagosome and lysosome vacuoles [2]. Initiation step is stimulated by physiological initiators such as oxidative stress or starvation, by pathological initiators such as excessive autophagic vacuoles, excessive aggregation of misfolded or unfolded proteins, bacteria, virus, *etc.* and by pharmacological initiators such as rapamycin [2]. Given that neurons are permanent post-mitotic cells, oxidative stress initiates the autophagy pathway and contributes to accumulation of the autophagic vacuoles and misfolded or unfolded proteins aggregates, and mutations in genes that encode the autophagic machinery proteins may lead to impairment of the entire autophagy pathway [10]. Impairment of at least one of the four steps of the autophagy pathway following sustained excessive oxidative stress, creates a state of continued activation of the deficient autophagic machine which can’t degrade the misfolded/unfolded proteins aggregates. Aging leads to a state of stress resulting from prolonged and excessive accumulation of the misfolded/unfolded proteins and autophagic vacuoles, which may contribute to neurodegeneration. Impairment of final step of maturation of autophagosome creates a stress state due to the impairment of lysosome fusion with autophagosome and of autolysosome formation [11]. In addition, autophagy is also known to play a role in the regulation of cell death and proliferation, in particular in models of heart and lung disorders, and autophagy may promote tumor-cell survival in cancer [2]. 

## 2. Air Pollution

Air pollution is among the most harmful environmental factors that causes cellular oxidative stress [12]. Air pollution has a very serious impact on public health all over the world. We have become accustomed to read about the relation between air pollution and pulmonary diseases [13]. Herein, we review the serious adverse effects of air pollution surpassing pulmonary diseases in the most common autophagy-mediated aging diseases such as neurodegenerative diseases, like Alzheimer’s, Parkinson’s, and Huntington’s diseases, amyotrophic lateral sclerosis (ALS) [14,15,16,17], and also Paget’s disease of bone [18]. According to Green Cross Switzerland and the Blacksmith Institute, there are regions affected by high atmospheric levels of polycyclic aromatic hydrocarbons (PAHs), heavy metals and other organic and inorganic compounds emitted by industries (lead, heavy metals), vehicle and wood heating smokes (particulate matter, heavy metals), and radioactive pollution from nuclear radiations (radioactive dust including uranium, plutonium, cesium-137, strontium-90) (Table 1) [19]. In some geographic area, human toxicity associated with exposure to high levels of air pollution have been documented (Table 1), but due to the limited life expectancy of inhabitants in these countries, no increase in prevalence of autophagy-mediated aging diseases has been documented yet. These components of air pollution such as heavy metals and PAHs are also present in most developed countries and can induce cellular oxidative stress and then contribute to multifactorial pathogenesis of common autophagy-mediated aging diseases. 

**Table 1:** The ten world’s worst polluted places.

Place/City	Country	Pollutant	Number of Affected Population	Known Health Risk	Reference
Agbogbloshie	Ghana	Lead	More than 40,000 persons		[20,21]
Chernobyl	Ukraine	Uranium, plutonium, and other metals	Up to 10 millions persons	Leukemia	[22]
Citarum River	Indonesia	Lead, cadmium, chromium, and pesticides	Up to 5 millions persons		[23,24]
Dzershinsk	Russia	Numerous chemicals	300,000 persons	Cancers of the eyes, lungs, and kidneys; Chronic cough with sputum	[25,26]
Hazaribagh	Bangladesh	Chromium	More than 160,000 persons	Acid burns, rashes, aches, dizziness, and nausea are also common health problems faced by local residents.	[27,28]
Kabwe	Zambia	Lead	More than 300,000 persons	Blood lead is more than 200 ug/dL in children (blood lead more than 120 ug/dL is fatal)	[29,30]
Kalimantan	Indonesia	Mercury, cadmium	More than 225,000 persons	Health risk for human, animals, and fish is that mercury in the Kahayan River of Central Kalimantan is 2260 ng/L.	[31]
Matanza Riachuelo	Argentina	Volatile organic compounds (VOCs) including toluene	More than 20,000 persons	Diarrheal diseases, respiratory diseases, and cancer	[32]
Niger River Delta	Nigeria	Petroleum	Non exactly known	Increase of the prevalence of childhood malnutrition. Crude oil leads to infertility and cancer because of its hemotoxic effect	[33]
Norilsk	Russia	Copper, nickel oxide, other heavy metals	135,000 persons	Respiratory diseases, and cancers of the lungs and digestive system	[34]

## 3. What Are the Main Air Pollutants Known to Impact the Oxidative Stress?

There is a very close relation between air pollution and oxidative stress [35,36]. Air pollutants released by heavy metals and PAHs work as catalysts in cellular redox reactions. Eventually, this leads to generation of ROS, which has a highly damaging role on DNA, RNA, cellular organelles and cellular proteins [37]. Oxidative stress is a stressful state in which highly energetic electrons can drain the cell contents if its energy isn’t well exploited by cellular organelles [38]. The imbalance of oxidant-antioxidant levels resulting from oxidative stress may lead to cell senescence, apoptosis or necrosis [39]. Autophagy is another important pathway that can attenuate the effect of the oxidative stress on the cell genetic material, cellular proteins, cellular components, and cellular organelles [40,41,42]. Impairment of the autophagy pathway may lead to accumulation of reactive oxygen species (ROS) and destruction of the main cellular component [42]. *SQSTM1/p62* gene, which is mutated in some cases of ALS, Paget’s bone disease, and frontotemporal dementia, encodes a protein that plays a crucial role in autophagy [43,44,45,46]. It works like a “cargo” to ubiquitin protein to eliminate the non-functional proteins by the autophagosome. Impairment of autophagy may contribute to the pathogenesis of some autophagy-mediated aging diseases [2].There are many sources of air pollution, such as fine particles, tobacco smoking, diesel exhaust exposure, that are associated with neurodegenerative diseases [47,48,49,50,51] and Paget’s disease of bone [52,53,54]. In this review, we will focus on four main sources of atmospheric pollution: residential wood heating, tobacco smoking, transportation and industry.

### 3.1. Residential Wood Heating

In fact, residential wood heating is a very serious problem of air pollution. For instance, Statistics Canada data show that there was a steady increase in the use of residential wood heating systems by approximately 60% from 1987 to 2000. Residential wood heating systems emit 44.1% of the fine particles emissions in the province of Quebec. Quebec Sources of Particulate Matter (PM_2.5_) in 2000 has shown that wood heating systems have a major role in the emission of fine particulates in Quebec. Residential wood heating systems account for approximately half of the PM_2.5_ rates in Quebec [55]. Residential wood heating systems also emit many organic or non-organic chemical pollutants, such as carbon monoxide, volatile organic compounds, acrolein, formaldehyde, and nitrogen oxides. Among the most harmful components are the following; PAHs, dioxins and furans, and fine particulates [55]. This fine particulate matter suspends in the air and can travel away for a long time as “air-borne pollutant”. Contents of the fine particulate matter are also dangerous because they contain sulfates, nitrates, carbon, organic substances, PAHs, ground minerals, and heavy metals. Finally, they can easily penetrate human tissues leading to cytokines activation or suppression, and to to side effects on the respiratory system, such as in asthma, pneumonia, bronchitis, emphysema or chronic respiratory diseases [56]. 

### 3.2. Tobacco Smoking 

Tobacco leaves contain many precursors that can be converted into other chemical compounds after ignition in high temperature during smoking. Polyphenols, carbohydrates, and trace metal ions are the main components of tobacco leaves, which can be converted into many compounds harmful to human health after pyrolysis, such as phenolic compounds, in addition to heavy metals. The complexity of tobacco smoke composition is due to the heating conditions of the lit cigarette. The temperature of the lit cigarette end ember may reach 900 °C. There are two types of cigarette smoke; mainstream is the smoke constituents drawn by the smoker during a puff, and sidestream refers to the smoke constituents escaping from the peripheral sides of the lit cigarette end [57]. Mainstream smoke contains concentrated components from the particulate phase (tar phase), which are droplets of many constituents suspended in a volatile gas phase. Using a fiberglass filter called Cambridge pad [57,58,59], scientists can easily trap the “particulate phase” (“tar phase” or “total particulate matter-(TPM)”) and gas phase. According to the Canadian Ministry of Health, the major contents of Canadian tobacco smoke are carcinogenic compounds, benzene, fine particulates, and hydrogen cyanide [57]. More than 4,000 chemicals result from tobacco smoking [60]. Harmful chemical carcinogenic compounds such as heavy metals (arsenic, cadmium, chromium, nickel, and lead), PAHs, benzene, organic compounds [61] have many harmful effects, principally on the immunological system. These heavy metals or PAHs can induce an oxidative stress at the level of cells leading to a continuous series of oxidative free radicals. These free radicals contribute later to destroying genetic and cellular contents such as DNA, RNA, and mitochondrial membranes.

### 3.3. Transportation and Industry

Weather from “rural emission” or from “urban emission”, transportation smog contains many organic and inorganic compounds such as carbon dioxide, methane, nitrous oxide, carbon monoxide, particulate matters, sulphur dioxide, oxides of nitrogen, sulphate aerosols, oxides of nitrogen, nitrate aerosols, ozone from NOx, benzene, ozone from volatile organic compounds (VOC), and non-methane VOC [62,63,64]. The main sources of heavy metals and PAHs emissions are industry, fossil-fuel combustion, transport, tobacco smoke (mainstream and sidestream), wood-heating smog, etc.

## 4. How Air Pollutants Play a Role in the Induction of Oxidative Stress?

We review in this part the components of cigarette smoke as an example of air pollution, and we will discuss its role in the induction of oxidative stress. Cigarette smoke have two different types of free radicals, one from tar phase “TPM phase” and the other from the gas phase [57,65], whereas other environmental air pollutants are known to contain a heterogeneous mixture of particles suspended in a liquid and gaseous phase [39].

### 4.1. Particulate-Phase Constituents

#### 4.1.1. Free Radicals 

Church and Pryor have identified more than 3000 compounds in the “tar phase free radicals”. Studies using electron spin resonance demonstrated that cigarette tar has many “stable quinone/hydroquinone (Q/QH_2_) radical species” which represent an active redox system that can reduce O_2_ to superoxide O_2_—then lead to formation of hydrogen peroxide (H_2_O_2_) and hydroxyl radical (OH), eventually leading to DNA damage [57,65]. Incubation of cigarette tar with DNA can create a new electron paramagnetic resonance spectroscopy signal that means cigarette tar components may create mutations in DNA [57]. Gas phase free radicals play a role as α1-protease inhibitor inactivation. α1-Protease inhibitor is a major serum antiprotease, an enzyme that plays a role in chronic obstructive pulmonary disease or emphysema. Gas phase free radicals may lead to oxidation of thiol-containing enzymes by producing NO and NO_2_. Thiol-containing enzymes play many important roles, in apoptosis such as caspase-9, and a role in chelation with heavy metals preventing their harmful action in the body such as metallothionein enzymes and matrix metalloproteinase 9 (MMP9), impairment of which may lead to highly deleterious complications [57,65,66].

#### 4.1.2. Quinones 

Toxicity of quinones occurs by a redox cycling mechanism, which lead to formation of excess amounts of ROS and quinones that deform the structures of some essential biological molecules by formation of covalent bonds with them [57].

#### 4.1.3. Heavy Metals

Heavy metals exist naturally in rocks and soil in some geographic area. Cadmium, mercury and lead are the most important harmful heavy metals to human health. Heavy metals can be transferred from the contaminated soil to plant by roots to leaves. They can also be transferred to plant leaves by acidic rains that result from industry fumes. One from these plants is tobacco, which is mainly consumed during cigarette smoking. By smoking tobacco leaves, heavy metals, especially cadmium, may transfer to humans by inhalation and last many years without excretion leading to long-term effects and many serious problems related to bone fragility, kidney diseases and lung damage [57,67]. 

### 4.2. Gas-Phase Constituents

#### 4.2.1. Oxidizing Radicals and Reactive Nitrogen Species (RNS)

Nitric oxide NO is one of the gas phase components of tobacco smoke. It is an oxidizing agent but a non-toxic and non-reactive radical. As soon as the vapor phase components containing it are in contact with air, NO·rapidly, within seconds, combines with the molecular oxygen O_2_ that normally exists in air. This combination leads to the formation of toxic nitrite ion NO_2_^·^ which is deemed a serious oxidant and nitrating agent. After a few seconds, NO_2_^·^ reacts with isoprene and butadiene present in the smoke to generate more serious highly reactive components called nitroso-carbon-centered radicals. This reaction between NO_2_· and tobacco smoke gas phase has also other consequences such as formation of other deleterious components called reactive nitrogen species (RNS). RNS include NO_2_, N_2_O_3_, and N_2_O_4_ and ONOO^-^ (peroxynitrite). These RNS have damaging effects on cell by nitration of the amino acid tyrosine [57,68,69,70]. 

#### 4.2.2. Peroxynitrite (ONOO^−^)

Peroxynitrite is not a free radical but it is a very strong oxidant of RNS, resulting from the reaction between two free radicals, nitric oxide anion and superoxide anion. In fact, O_2_^‒^ results from the “particulate phase”. Superoxide dismutase competes with NO· to react with O_2_. If NO· dominated, the reaction will happen resulting in peroxynitrite. The harmful effect of O_2_^−^ is due to formation of peroxynitrite that leads to a very powerful disruptive action on cellular contents. The serious effects of peroxynitrite lie in its reactions and suppression of many important vital proteins such as hemoglobin, myeloperoxidase, glutathione peroxidase [57,71].

#### 4.2.3. Glutathione Depleting Substances 

Many electrophile constituents distinguish cigarette gas phase. These electrophilic constituents search for and attract electrons from other compounds such as thiol group-containing compounds like glutathione. Gas phase electrophile components‒thiol group-containing compounds reaction leads to changes in different proteins and in cellular chemistry. One of the serious results of gas-phase electrophile components-thiol group-containing compounds reaction is glutathione depletion. Glutathione exists in the mitochondria, in nuclei, and in the cytoplasm, and glutathione is also an antioxidant that plays many essential cellular functions [57,72]. 

## 5. Air Pollutants and Oxidative Stress in Common Autophagy-Mediated Aging Diseases

Among the air pollutants, tobacco is probably the best documented one for its direct impact on autophagy, in particular in the lungs, but also in organs without any direct contact with tobacco smoke. For instance, exposure to cigarette smoke was reported to induce a dysfunction of mitochondrial repair mechanisms, leading to autophagy-mediated follicle death [73]. In addition, exposure to air pollution particulate matter with a diameter less than 2.5 μm also called PM_2.5_, which is known to induce oxidative stress, was also recently reported to induce autophagy in human lung epithelial cell lines and thus contribute to impaired pulmonary function [74]. There are three major cell death pathways: apoptosis, autophagy and necrosis [75]. Autophagy, which is one of the three types of programmed cell death with apoptosis and non-lysosomal cell death [76], was reported to be impaired in cancer and many degenerative diseases. In addition to autophagy, other cellular process such as necrosis, ROS-dependent or ROS-independent apoptosis, have been involved in air pollution cell death [39]. Alzheimer’s, Parkinson’s, Huntington's diseases, and amyotrophic lateral sclerosis (ALS), frontotemporal dementia (FTD), and inclusion body myopathy with Paget’s bone disease and/or frontotemporal dementia (IBMPFD) are examples of diseases affected by autophagy impairment (Table 2). In familial forms of Alzheimer’s disease, mutations in *Amyloid precursor protein (APP), Persenilin-1 (PSEN1)*, and *Presenilin-2 (PSEN2)* genes lead to excessive formation of neurotoxic peptide amyloid- β (Aβ) plaques which aggregate extracellularly. Hyperphosphorylated Tau protein aggregates within the cell, with neurofibrillary tangles leading to excessive intracellular aggregations. α-synuclein oligomers and Aβ aggregation and accumulation in the mitochondrial membrane leads to formation of cytochrome C and caspase cascade activation contributing to Alzheimer’s disease pathophysiology [77,78]. In Parkinson’s disease, causal mis-sense mutation in *α-synuclein* and mutations in *Leucine-rich repeat kinase 2 (LRRK2, PARK8)* genes have been identified. Mutation in *α-synuclein* gene leads to Lewy bodies formation, that are accumulated in excessive aggregations. Difficulty in clearance these bodies leads to Parkinson’s disease [11,79]. In Huntington's disease, a mutant *Huntington* (*Htt*) gene has more than 35 repeats of the triplet CAG (cytosine-adenine-guanine) and encodes a mutant Htt protein that forms toxic oligomers or insoluble aggregates. These insoluble mutant Htt protein aggregates are neurotoxic and lead to Huntington’s disease. Autophagy is responsible for removing and recycling this mutant non-functional protein. In case of impairment autophagy, by an environmental factor such as a sustained excessive action of oxidative stress, Huntington’s disease show aggravated symptoms and will be more difficult to control [80]. In familial ALS, 15% of patients have *Superoxide dismutase1* gene mutations and 3% of patients have rare variants in *TDP-43* or *FUS/TLS* genes. Excessive formation of abnormal levels of mutant proteins aggregation makes the autophagic process less efficient. Accumulation of these mutant proteins and deficiency of the autophagy in removing and recycling these non-functional proteins lead to neurodegeneration and ALS symptoms [81,82,83]. In FTD, mutations in *Valosin Containing Protein (VCP)/p97*, and/or mutations in the ESCRT-III subunit of the *CHMP2B* gene may also lead to formation of mutant non-functional proteins aggregation. Excessive and sustained accumulation of these proteins aggregation bodies, if not well removed by the autophagy process, contribute to neurodegeneration and FTD symptoms [84,85,86]. In IBMPFD, bone, muscle, and brain are the three affected tissues. VCP/p97 plays a very crucial role in formation of autophagosomes. Mutations in the *VCP* gene have an impact on the maturation of autophagosomes leading to immature autophagosomes or inclusion bodies. Aging by allowing an excessive accumulation of intracellular immature autophagosomes or intracellular inclusion bodies and the presence of oxidative stress by increasing the impairment state of the autophagy participate in the etiology of IBMPFD syndrome [11,83,87].

**Table 2:** Main molecular bases of autophagy defect in the most common autophagy-mediated aging diseases.

Disease	Main Molecular Characteristics on the Autophagy Defect Leading to an Accumulation of Autophagy Vacuoles	Reference
Amyotrophic Lateral Sclerosis	Mutant SOD1 and/or rare variants in TDP-43 or FUS/TLS genes	[82,83]
Alzheimer disease	Assembly of amyloid-β (Aβ) peptide and hyperphosphorylated Tau protein intracellular tangles Defect in clearence of amyloid-β (Aβ) peptide	[77,78]
Frontotemporal Dementia	Mutant CHMP2B (ESCRT-III subunit) and/or Mutant p97/VCP Defect in maturation of autophagosome and in the fusion of autophagosome and lysosomes	[85,86]
Huntington’s disease	Aggregation of oligomers of mutant Htt protein	[80]
IBMPFD syndrome	Mutant p97/VCP Inclusion bodies formation Defect in maturation of autophagosome, in fusion of autophagosome and lysosomes, and in clearance of inclusion bodies	[83,87]
Paget’s disease of bone	Impairment of the autophagic flux due to mutant p62/SQSTM1	[88]
Parkinson’s disease	Mutant α-synuclein protein Lewy bodies formation Defects in clearance these bodies	[11,79]

Paget’s bone disease, the second most frequent metabolic bone disorder after osteoporosis, is also affected in the same autophagy pathway by functional impairment of SQSTM1/p62 as a consequence of *SQSTM1* gene mutations and proteasomal pathway impairment [16,88]. Microautophagy, corresponding to invagination with lysosomal vacuoles, is one of the three types of autophagy processes. The second one is the chaperon-mediated autophagy in which proteins marked by a “pentapeptide motif” (KFERQ), undergo lysosomal degradation. Thirdly, macroautophagy (called here “autophagy”) that is distinguished by the formation of autophagosomes. Phagosomes are double membrane bodies that swallow malformed, misfolded, non-functional proteins or organelles [76,89]. Activation of PI-3 kinase/mTOR pathway or non-functional beclin-1 which are involved in autophagy processes, may lead to tumors [9,42]. In tumor cells, Mathew *et al.* have highlighted the cell life rescuer role of autophagy in case of functional apoptosis and non-functional apoptosis. As a result, scientists have discussed the role of autophagy inhibition in rapid elimination of cancer cells [41]. Damaged non-functional proteins or misfolded proteins result from stress (by stress oxidation or by starvation, *etc.*) undergo a process of “refolding”, aggregation and ubiquitination. Cells try to get rid of these non-functional proteins by the proteasome pathway or by the autophagy pathway. In autophagy, ubiquitinated proteins will be directed by SQSTM1/p62 protein to the autophagosome. Aggregated “polyubiquinated” non-functional proteins swallowed by the autophagosome after binding to Atg8/LC3 that present at the surface of the autophagosome. Consequently, non-functional proteins are eliminated from the cell by lysosomal action [9]. In Paget’s bone disease, the deficiency of SQSTM1/p62 results in an abnormal autophagy process leading to the accumulation of aggregated ubiquitinated proteins, which induce osteoclastogenesis by activation of the NF-kB pathway [88]. 

## 6. Oxidative Stress in Bone Remodeling

Free radicals are radicals produced in the chemical, biological medium (*in vitro*) or in biological organs (*in vivo*) by some biological reactions, which can destroy cellular membranes and can also create mutations in DNA or histones [90]. Free radicals of oxygen and of nitrogen are a notable example of biologically harmful free radicals [90,91,92]. Many type of tumors, obesity, diabetes, and other metabolic disorders are associated with an excessive number of free radicals [93]. Normally some cellular enzymes such as superoxide dismutasecan neutralize free radicals produced in the biological medium. In case of excess numbers of free radicals, cells can’t afford to continue neutralizing this huge number of free radicals, which leads to an imbalance between oxidants and antioxidants in the cell [94]. ROS are also normally produced during oxidative phosphorylation; the process that occurs in the inner mitochondrial membrane, which also aims to produce energy [95]. In the electron transport chain, electrons undergo a series of oxidation-reduction reactions and cross through many proteins and enzymes. In abnormal conditions, oxygen is incompletely reduced, giving superoxide radical anion O_2_^−^. Large amounts of O_2_^−^ may damage the mitochondrial proteins and damage the mitochondria itself [96]. The protein B-cell lymphoma/leukemia 2 (Bcl-2) [97], which is very sensitive to any damage in mitochondrial content, activates Bax proteins which in turn open pores in the mitochondrial membrane inducing cytochrome C to get out of the mitochondrial membrane and bind to apoptotic protease activating factor-1 (Apaf-1) which is present in the cytosol. Apoptosome is rapidly formed by binding of Apaf-1 with the cytochrome C. This complex in turn activates caspase-9 which denatures the mitochondrial membrane leading to mitochondrial death and the cell phagocytosis [98,99]. Increased levels of ROS increase signaling through mitochondria-associated antiviral receptor which increases Interferon Regulating Factors-3 (IRF-3), and IRF-7 and NF-kB leading to the antiviral action [100]. In osteoporosis, a negative correlation between bone mineral density and oxidative stress, has been found [101]. Kyung *et al.* discovered that antioxidants such as rutin decrease bone density by inhibiting osteoclast formation by inhibiting NF-κB activation [102]. Wauquier *et al.* have shown that ROS stimulate RANKL-induced osteoclastogenesis, inducing bone loss by apoptosis and by decreasing differentiation and activities of osteoblasts [103]. High levels of ROS are the cause of oxidative stress which deregulates redox-sensitive transcription factor activities. Among redox- sensitive proteins involved in bone differentiation, are mitogen-activated protein kinases; Wnt/Beta catenin; and NF-kB. MAPKs such as extracellular signal-regulated kinase (ERK1/2), P38 and c-jun N terminal kinase (JNK), also protein kinase A (PKA) and cAMP response element-binding protein (CREB) have a close relation with ROS activities in bone cells. According to Bai *et al.* ROS can induce ERK-dependent NF-kB pathway to reduce osteoblastogenesis while inducing osteoclastogenesis [104]. The expression of P38 which is involved in both osteoclast and osteoblast formation can be increased by ROS leading to increasing osteoclastogenesis, while in osteoblasts, ROS inhibit p38 leading to lowering osteoblast formation [103]. Studies by Lee *et al.* have confirmed the effect of ROS on MAPKs. Following inhibition ROS production by Nox-1, JNK, P38 and ERK 1,2 were inhibited, which represent promising avenues for future targeted therapies [105]. These data demonstrate that ROS (O- and H_2_O_2_) can promote osteoclastogenesis by PKA-CREB and ERK pathways, by increasing RANKL expression in bone cells [104].

## 7. Conclusions

The oxidative stress and ROS and/or RNS formation, which can result from exposure to environmental factors such as air pollutants, may lead to impairment of the autophagy process, which may possibly contribute into aging disorders such as neurodegenerative diseases and Paget’s bone disease. Further studies are required to determine the role of oxidative stress resulting from air pollution on autophagy-mediated aging disease pathogenesis.
